# Editorial: Management of borderline ovarian tumor: The best treatment is a real challenge in the era of precision medicine

**DOI:** 10.3389/fsurg.2023.1167561

**Published:** 2023-03-07

**Authors:** Luigi Della Corte, Valeria Cafasso, Antonio Mercorio, Giuseppe Vizzielli, Carmine Conte, Pierluigi Giampaolino

**Affiliations:** ^1^ Department of Neuroscience, Reproductive Sciences and Dentistry, School of Medicine, University of Naples Federico II, Naples, Italy; ^2^ Department of Public Health, University of Naples Federico II, Naples, Italy; ^3^ Clinic of Obstetrics and Gynecology, “Santa Maria della Misericordia” University Hospital – Azienda Sanitaria Universitaria Friuli Centrale, Udine, Italy; ^4^ Department of General Surgery and Medical-Surgical Specialties- Institute of Obstetrics and Gynecology, A.O.U. Policlinico Rodolico-San Marco, University of Catania, Catania, Italy

**Keywords:** bordeline ovarian tumor, conservative surgery, fertility, recurrence, survival

**Editorial on the Research Topic** Management of borderline ovarian tumor: The best treatment is a real challenge in the era of precision medicine

## Introduction

Borderline ovarian tumors (BOT) are a common neoplasm of the female reproductive system, accounting for 10%–15% of all epithelial ovarian cancers (OC), showing atypical epithelial proliferation without stromal invasion. They are typically diagnosed at an early stage while still confined to the ovaries, usually during reproductive age, and before 40 years old (Raimondo et al.; Wang et al.). About 50% of BOTs are of the serous histotype (sBOT), followed by the mucinous type (mBOT) (Wang et al.; Gaballa et al.; Della Corte et al.). In addition, sBOT is bilateral in 15%–25% of patients and it can spread to the peritoneum in up to 40% of cases, with a higher risk of relapse ranging from 7.8% to 34% (Raimondo et al.; Della Corte et al.). mBOTs are almost always unilateral and large in size, with a lower risk of recurrence compared to sBOTs, but in the event of a relapse the risk of invasive disease is higher (Della Corte et al.). Patients’ symptoms are nonspecific, such as vague abdominal pain/discomfort and an abdominal mass, or urological involvement (Gaballa et al.). They are staged according to the FIGO system established for OCs. Patients with BOTs have a good prognosis, with a 5- and a 10-year overall survival (OS) rate of 95%–97% and 90% for women with FIGO stages I–III, respectively, while the OS decreases to nearly 77% in the case of stage IV (Raimondo et al.; Della Corte et al.). Surgical treatment is the main option for BOTs, and it depends on preoperative diagnostic features, histologic type, tumor stage, and fertility desire. Indeed, a fertility-sparing approach can be opted for by young patients with stage I as an alternative to radical treatment. It generally consists of unilateral/bilateral cystectomy, unilateral or bilateral salpingo-oophorectomy, with preservation of the uterus. Although to date there is no global consensus on recommending hysterectomy for the treatment of BOTs, radical treatment has long been the gold standard and it includes hysterectomy with bilateral salpingo-oophorectomy (Raimondo et al.; Gaballa et al.; Della Corte et al.) and omentectomy (Della Corte et al.). The recurrence rate after radical surgery is significantly lower than that after fertility-sparing surgery (0%–5% vs. 13%, respectively), although it has been documented that the recurrence rate does not affect overall survival (Wang et al.). The decision to perform the lymphadenectomy must be evaluated individually, although it is usually recommended in cases with suspicious lymph nodes (Gaballa et al.; Della Corte et al.).

## Results

In this issue of Frontiers in Surgery titled “Management of borderline ovarian tumor: the best treatment is a real challenge in the era of precision medicine” we gathered five publications that describe the whole management and treatment of BOTs. Of these five papers, four are original articles (Raimondo et al.; Wang et al.; Gaballa et al.; Niu et al.), and one is a review (Della Corte et al.).

In preparing this volume, the editorial team sought to highlight the management of BOTs, particularly in younger patients. It is well known that BOTs have variable biological characteristics in addition to heterogeneous clinical behavior, so it is difficult to diagnose them preoperatively; moreover, their prognosis is strongly related to the surgical approach chosen.

Gaballa et al. evaluated the management and follow-up of BOTs in a tertiary referral center in Egypt; they showed how patients with mBOTs were significantly younger (40.083 ± 18.47 vs. 53.73 ± 11.91 years, *p* = 0.028) and with CA125 levels significantly higher in mucinous than serous and seromucinous types [67 (16–304) vs. 20 (6–294.6) U/ml, *p* = 0.027] (Gaballa et al.). Niu et al. found increased CA19-9 serum levels (52/221 cases, 23.5%) in women with mBOTs, while no differences in CA125 levels were noted among tumor types (Niu et al.). Both concluded that, although the specificity of CA125 was not very high, it could still sensitively determine the extent of disease involvement.

The ultrasonographic features of BOTs include solid cystic masses with mostly regular morphology, as reported by Niu et al. Typically, in cases of sBOTs, visible septa are present, many with visible blood flow; additionally, most of them have papillae (28 cases—63.6%), and small papillae may appear on the septations. This “microcapsule pattern” is an ultrasonic marker unique to borderline tumors. Indeed, a papillary protrusion is a typical feature of sBOT (Wang et al.). Conversely, the mucinous type is more frequently associated with the presence of septations (69 cases—77.5%) and is larger in size. However, because OC has similar ultrasound patterns to BOTs, it is difficult to accurately differentiate between the two; supposedly, the risk of malignancy increases with irregularity of wall and papillae, increasing size of papillae, irregular shapes, unclear borders and complex cystic (Wang et al.; Niu et al.). Frozen section (FS) can be used to obtain a preliminary diagnosis and to establish appropriate surgical management, although the diagnostic accuracy rate of FS seems to be rather high for benign and malignant ovarian tumors, unlike sBOTs, where it is rather low; in fact, it is classified as benign in 25%–30% of cases and as malignant in up to 30% of cases (Gaballa et al.; Della Corte et al.).

Regarding the possibility of recurrence, elevated CA125 levels, large tumor size, and cystic solidity on ultrasonography are clinically significant in predicting tumor relapse; so, these patients should undergo comprehensive staging surgery and close monitoring (Niu et al.). Some additional elements, such as micropapillary pattern, stromal microinvasion, stage IC3, and grading 3, may also be used to identify patients at risk of invasive recurrence. Microinvasive BOTs recurred at a higher rate and at a younger age than noninvasive BOTs, 17.4 vs. 7.8% (OR: 3.55, 95% CI: 1.091–11.59, *p* = 0.03) and 10.5 vs. 17 months respectively (Della Corte et al.). Della Corte et al. also reported that the recurrence rate is time-dependent, and relapse is more common in the first two years of follow-up after surgery. However, relapses can occur up to 15 years later, necessitating long-term follow-ups (Della Corte et al.). Levels of CA125 can be used for postoperative monitoring in association with transvaginal ultrasound (Della Corte et al.; Niu et al.).

As mentioned above, a large number of BOTs involve women of childbearing age when fertility preservation is desired, so conservative treatment is recommended in these patients. Unilateral cystectomy or salpingo-oophorectomy is considered the main line of treatment as a fertility-sparing approach, although a higher recurrence rate has been documented in these patients (up to 25%), but without affecting OS (Gaballa et al.). In terms of histology, unilateral salpingo-oophorectomy is considered the preferred surgical treatment in case of mBOTs, due to the high risk of invasive recurrence, whereas cystectomy is recommended mainly in cases of bilateral mBOTs or in patients who have previously had salpingo-oophorectomy to preserve fertility. Unilateral salpingo-oophorectomy rather than cystectomy does not appear to prevent relapses in microinvasive BOTs. Finally, in cases of bilateral ovarian involvement, mainly if serous subtype, bilateral cystectomy would appear to improve fertility outcomes without increasing the recurrence rate if compared with unilateral salpingo-oophorectomy and contralateral cystectomy (Della Corte et al.).

Because of the increased risk of extraovarian recurrence in such cases, radical surgical treatment should be considered in those patients who have fulfilled their desire for motherhood (Gaballa et al.; Della Corte et al.). As previously stated, radical treatment consists of hysterectomy with bilateral salpingo-oophorectomy, while the role of the uterine preservation has been poorly investigated. In this regard, Raimondo et al. evaluated the oncologic outcomes of postmenopausal women with BOT who underwent uterine-sparing surgery without ovarian preservation, as compared with patients undergoing hysterectomy, thereby determining the impact of uterine preservation alone on survival outcomes. The disease-free survival (DFS) and OS rates of the two treatment groups did not differ significantly, highlighting how the increased risk of recurrence reported for fertility-sparing treatment may be influenced by ovarian rather than uterine preservation. In addition, no uterine recurrence of BOT was found, which is consistent with what has been reported in the literature, where uterine recurrence is defined very rarely. Therefore, considering the lack of impact on oncologic outcomes hysterectomy could be avoided in the surgical staging of BOT patients, owing to the increased time, cost, and complexity of the procedure (Raimondo et al.).

Another important issue concerns the diagnosis and treatment of BOTs during pregnancy, as demonstrated by Wang et al. Clinical manifestations most frequently presented by patients were cysts, cyst enlargement, or cyst rupture. Among oncological markers, CA199 and CEA can be useful for diagnosis, monitoring, and follow-up because they are not affected by a patient's pregnancy status, unlike CA125, which can fluctuate during pregnancy. In comparison to postoperative definitive histology, FS has a low coincidence rate outside of pregnancy. In terms of treatment, conservative surgery appears to be safe and effective during pregnancy; in fact, maternal outcomes are as good as child outcomes. Because of the possibility of recurrence, even after a long period of time, long-term follow-up is required.

## Summary

Fertility-sparing surgery is a well-established strategy available for young patients with BOTs who want to preserve their fertility, with excellent reproductive outcomes and long-term survival. Uterine-sparing surgery during BOT surgical treatment did not lead to a significantly increased risk of recurrence and could be avoided in surgical staging in order to reduce the complexity, time, and cost of surgery.

Lastly, in patients with BOTs, oncologic markers, and ultrasound features may be helpful tools both before and after surgery.

